# Burden of allergic respiratory disease: a systematic review

**DOI:** 10.1186/s12948-016-0049-9

**Published:** 2016-09-28

**Authors:** A. Linneberg, K. Dam Petersen, J. Hahn-Pedersen, E. Hammerby, N. Serup-Hansen, N. Boxall

**Affiliations:** 1 Research Centre for Prevention and Health, The Capital Region of Denmark, Copenhagen, Denmark; 2 Department of Clinical Experimental Research, Rigshospitalet, Copenhagen, Denmark; 3 Department of Clinical Medicine, Faculty of Health and Medical Sciences, University of Copenhagen, Copenhagen, Denmark; 4 Department of Business and Management, Faculty of Social Sciences, Aalborg University, Aalborg, Denmark; 5 ALK, Hørsholm, Denmark; 6 MAPI, London, UK

**Keywords:** Allergic rhinitis, Allergic asthma, Economic burden, Meta-analysis, Systematic review, Quality of life

## Abstract

This meta-analysis compared the health-related quality of life (HRQL) of patients with allergic rhinitis (AR) and/or allergic asthma (AA) caused by perennial house dust mite (HDM) versus AR and/or AA caused by seasonal pollen allergy. Following a systematic search, the identified studies used the disease-specific rhinitis quality of life questionnaire or generic instruments (SF-36 and SF-12). Summary estimates obtained by meta-analysis showed that HRQL in patients with perennial HDM allergy was significantly worse than that of patients with seasonal pollen allergy, when measured by both disease-specific and generic HRQL instruments, and was reflected by an impact on both physical and mental health. A systematic review of cost data on AR and AA in selected European countries demonstrated that the majority of the economic burden was indirectly caused by high levels of absenteeism and presenteeism; there was little or no evidence of increasing or decreasing cost trends. Increased awareness of the detrimental effects of AR and/or AA on patients’ HRQL and its considerable cost burden might encourage early diagnosis and treatment, in order to minimize the disease burden and ensure beneficial and cost-effective outcomes.

**Electronic supplementary material:**

The online version of this article (doi:10.1186/s12948-016-0049-9) contains supplementary material, which is available to authorized users.

## Background

In Europe, approximately 23 % of the population is affected by allergic rhinitis (AR) [1]. The most common symptoms are sneezing, itchy nose, rhinorrhea, and/or nasal congestion [2], with many patients experiencing symptoms of at least moderate severity; one study estimated that 93 % of patients with AR who consulted general practitioners had symptoms that were moderate-to-severe [3]. Studies of patients consulting general practitioners for AR reported that 18–48 % had symptoms that were not controlled by pharmacotherapy [4, 5]. Despite the bothersome nature of symptoms, AR is often trivialized by the patient; less than half (45 %) seek medical advice or treatment for their condition [4], which results in under-treatment and poor control of symptoms. A UK study found that only 18 % of patients with AR had consulted their doctor about the condition in the preceding 2 years [6]. In a sample of 230 French patients with typical symptoms of AR, 19 % had never consulted their doctor about the problem, despite 90 % reporting that their nasal symptoms affected their daily lives, over half complaining of sleepiness and headaches, and 8 % reporting that they had taken time off work because of their rhinitis [7]. Allergic rhinitis is frequently accompanied by allergic asthma (AA), the prevalence of which increases from less than 2 % in individuals without AR to 10–40 % in those with AR; this combination of upper and lower respiratory symptoms increases the overall impact on the patient [8–10].

There is now considerable evidence that the symptoms of AR and AA negatively affect patients’ health-related quality of life (HRQL) [11–20]. This is supported by evidence that successful treatment of symptoms with allergy immunotherapy (AIT) [21–23] or symptomatic treatments [24–31] improves the HRQL of patients with AR. These improvements generally mirror those of conventional clinical outcome measures, such as symptom and medication scores, and lung function tests [32]. Although clinical measures provide information on the affected organ systems, they do not capture the patient’s overall perception of the disease burden caused by the physical, emotional, and social impairments in everyday life [33]. The wider impact of AR on patients’ lives is now acknowledged in the allergic rhinitis and its impact on asthma (ARIA) guidelines, which recognize the impact of HRQL on AR, and classify its severity based both on symptoms and its effect on HRQL [32].

There is evidence that AR represents a considerable economic burden [34, 35], with annual costs in the US in 2003 estimated at $2–$5 billion USD [34]. Indirect costs resulting from symptoms causing under-performance or loss of productivity in the workplace were particularly high [36–39]. Data published after the present study was completed show that patients with mild AR have less impact on the health economy, with costs of around a quarter of those with moderate-to-severe disease [39].

To date, there has been no systematic review of HRQL data in patients with perennial or seasonal allergies. The objective of this systematic review and meta-analysis was, therefore, to compare HRQL in individuals with AR and/or AA with perennial house dust mite (HDM) or seasonal AR caused by exposure to pollen, or both. As HRQL instruments/questionnaires are included in many clinical studies as a matter of course, HRQL measurements enable comparisons with data from individual studies. In addition, data on the economic burden of AR and AA in selected European countries were systematically reviewed in order to compare direct and indirect costs in patients with perennial HDM and seasonal AR.

## Methods

### Search and identification of studies

To identify published articles on HRQL in individuals with AR and/or AA, and the economic burden of AR and AA, the following online databases were searched: MEDLINE and MEDLINE In-Process (1946–31 January 2014); EMBASE (1988–2014 week 04); EconLit (1886–January 2014); The Cochrane Library (Cochrane Central Register of Controlled Trials and Cochrane Database of Systematic Reviews); Database of Abstracts of Reviews of Effects; National Institute For Health Research Health Technology Assessment Programme databases; Health Economics Evaluation Database; National Guidelines Clearing House; NHS Evidence; EUROScan; and Clinicaltrials.gov.

The search strategies and keywords used are presented in the Additional file 1. The MEDLINE, EMBASE, EconLit, Cochrane Library, Database of Abstracts of Reviews of Effects, and NIH HTA databases were searched simultaneously using OVID. Unless otherwise stated, the search covered the period from 1 January 2000–31 January 2014 (date of publication).

### Study selection

Articles considered for inclusion included randomized, controlled trials, observational and ecological studies, and health economic analyses with HRQL and/or cost as a primary or secondary outcome from one or more of the eight countries of interest: Denmark, France, Germany, Italy, the Netherlands, Spain, Sweden and the United Kingdom. Eligible studies enrolled adults or children diagnosed with (based on positive skin prick tests and/or specific IgE results), and/or treated for, HDM (*Dermatophagoides pteronyssinus* or *Dermatophagoides farinae*) allergy; allergy to grass pollen (orchard, meadow, ryegrass, sweet vernal, and timothy grasses), *Artemisia* pollen, ragweed pollen, or birch pollen; or AA (as defined in the individual studies). Studies of AIT and symptomatic treatments were eligible for inclusion.

Titles and abstracts identified by the literature search were reviewed by NB; a second senior epidemiologist assessed 10 % of the titles and abstracts, and any disagreements were resolved through discussion. Full text versions of potentially eligible studies were obtained and reviewed. Additional relevant articles were identified from the bibliography of some articles, and a report from a clinical trial containing unpublished data was provided by the sponsor. Titles and abstracts of the 20 highest ranked Google Scholar search results were also reviewed and compared with the results of the database search to ensure that no articles had been missed.

### Data extraction and meta-analysis

As the aim of this study was to gather information on the HRQL of patients before any intervention, baseline measurements of HRQL (prior to any intervention) in patients with AR and/or AA were extracted from the selected studies. Means and standard deviations (SD) were collected, or where this was not possible, were imputed from available statistics, according to the methods described in [40]. Where data were presented as p < 0.05 or p < 0.01, values used for imputations were p = 0.049 and p = 0.009, respectively. Six studies presented data graphically, and values were obtained using a ruler.

Meta-analyses were performed in Microsoft Excel, and heterogeneity was assessed using Q-tables [41]. Differences between subgroups were assessed by ANOVA [42]. Due to the expected differences in study design, random effects models were used to estimate the overall measure of HRQL and the error of the estimate. Pooled mean estimates were presented with standard errors and 95 % Confidence Intervals (CI).

Where possible, HRQL data were grouped by allergen (perennial or seasonal); if the allergen was not stated, data were included in a ‘Mixed’ group. Some older articles did not specify the sensitizing allergen but categorized patients as suffering from seasonal AR (SAR) or perennial AR (PAR); in these cases PAR was grouped with HDM allergy, and SAR with pollen allergy.

### Cost data

Cost data were divided into direct costs (including physician visit, medication costs, hospital stays, comorbidity medication costs, comorbidity physician visits, emergency room visits, acute ward visits, and outpatient care) and indirect costs related to loss of productivity (including absenteeism, presenteeism, production loss, inability to perform usual daily activities, restricted usual daily activities). All costs were converted to 2013 international dollars ($), and expressed as purchasing power parities (PPP).

## Results

### Search results and selected studies

**Fig. 1:**
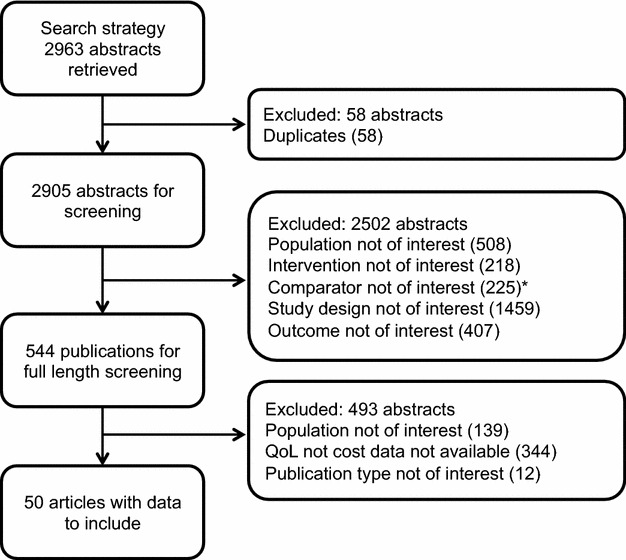
Schematic diagram of the selection process. *Asterisk* comparator not of interest = environmental control mechanisms, such as mattress protector or humidifier

The electronic search retrieved 2963 abstracts and titles; 2905 were retained for screening, and 2502 of these did not meet the inclusion criteria. Full text copies of 544 articles were screened and 50 were retained for data extraction. One additional reference was identified from the reference section of an article [43] and data were included from an unpublished study report provided by the sponsor [44]. Flow of articles through the selection process and reasons for rejection are shown in Fig. 1. Among the studies reporting HRQL data, 3 included patients with AA; two with PAR+AA, and one with SAR+AA.

### Baseline HRQL measurements: RQLQ and mini-RQLQ

The disease-specific rhinitis quality of life questionnaire (RQLQ) is a validated HRQL instrument for adults that measures functional impairments due to seasonal or perennial rhinoconjunctivitis of allergic or non-allergic origin. It comprises seven domains scored on a 7-point scale (from 0 = not impaired at all, to 6 = severely impaired), with higher scores reflecting poorer HRQL [45, 46].

Seventeen studies reported data obtained using RQLQ or mini-RQLQ measured at ‘baseline’, ‘the season before’ or on ‘a day without symptoms’ [5, 24, 43, 47–60]. One study included patients with SAR/pollen allergy only (n = 83) and SAR/pollen allergy with associated AA (n = 52) [49]. Two studies [59, 60] contained data that did not correspond to the recognized RQLQ scale and were excluded from the analysis.

**Fig. 2:**
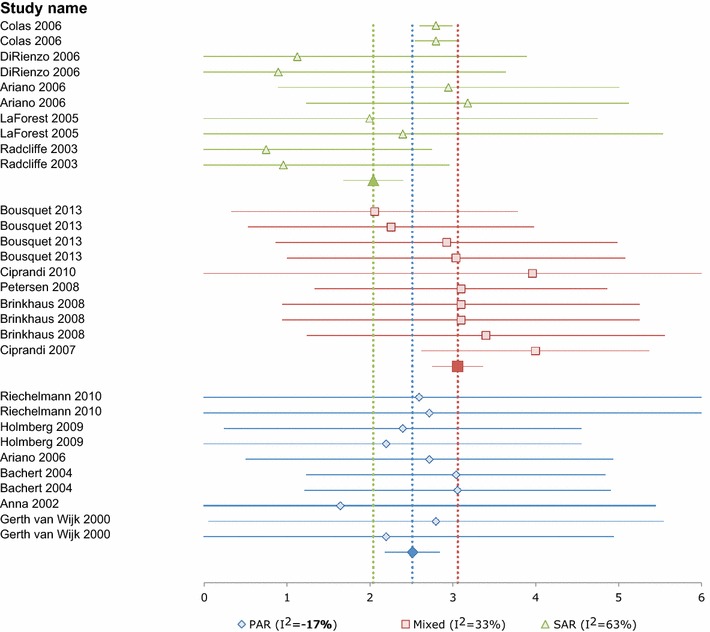
Forest plot of overall RQLQ baseline scores by phenotype, from 30 groups in 15 studies. Higher scores indicate poorer HRQL

Pooling all the data gave a moderate degree of heterogeneity (I^2^ = 62 %) with an estimated summary RQLQ of 2.61 ± 0.10 (95 % CI 2.43–2.80). Grouping studies by allergy phenotype [SAR/pollen only, PAR/HDM only, or SAR/pollen and/or PAR (‘Mixed’ group)] gave I^2^ values of 63 % (moderate) for SAR/pollen, 33 % (low) for ‘Mixed’ group, and −17 % (none) for the PAR/HDM groups. Summary RQLQ estimates for the respective groups were 2.04 ± 0.18 (95 % CI 1.68–2.41) for the SAR/pollen patient group (673 patients in 10 groups from 5 studies); 3.06 ± 0.16 (95 % CI 2.75–3.37) for the Mixed group; and 2.73 ± 0.12 (95 % CI 2.49–2.98) for the PAR/HDM only group (1520 patients in 10 groups from 6 studies) Fig. 2.

### Baseline HRQL measurements: SF-36 and SF-12

The 36-Item Short Form Health Survey (SF-36) is a validated, non-disease-specific indicator of overall health status. It comprises 36 items in eight domains that reflect physical and mental functioning, which are scored from 0 to 100, with a lower score indicating poorer HRQL [61]. The SF-12 instrument is a short form of the SF-36 questionnaire, comprising 12 items.

Seven studies reported data using SF-36 or SF-12 [13, 14, 49, 52, 62–64]. One study included patients with SAR/pollen allergy only (n = 83) and SAR/pollen allergy with associated AA (n = 52) [49], and one included PAR/HDM patients with AR (n = 85), AR + AA (n = 62) or rhinitis, asthma and atopic eczema/dermatitis syndrome (n = 31) [63]. Data from one study [64] were insufficient to calculate SDs and this study was excluded from the analysis.

**Fig. 3:**
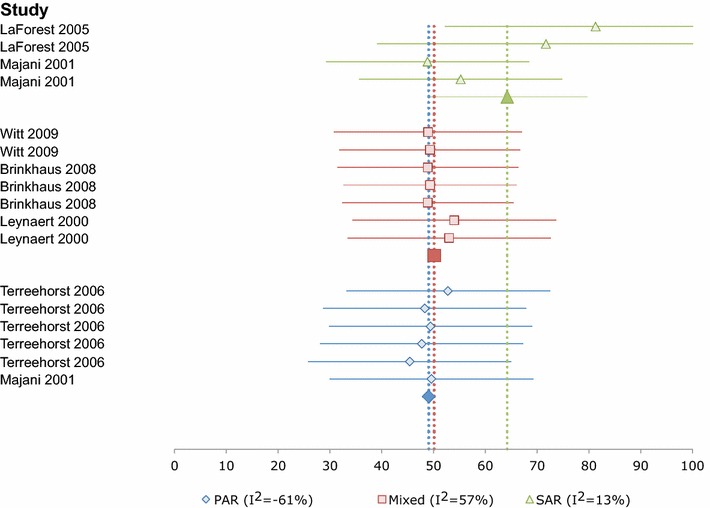
Forest plot of baseline SF-36/SF-12 physical component scores from 17 groups in six studies by phenotype. 95 % confidence intervals for the Mixed and PAR summary measures are hidden by the *symbols*. Lower scores indicate poorer HRQL

Data for the two main SF-36 composite domains were analyzed, to investigate whether the effects of allergic respiratory disease on physical and mental HRQL differed. Heterogeneity was high when all SF-36 physical component score (PCS) data were pooled (I^2^ = 84 %). When data were grouped by phenotype, there was no heterogeneity for SAR/pollen (I^2^ = 13 %) or the PAR/HDM group, (I^2^ = −61 %), and heterogeneity was low for the Mixed group (I^2^ = 57 %). Summary estimates of HRQL using the SF-36 PCS for respective phenotypic groups were: SAR/pollen group 64.19 ± 7.89 (95 % CI 48.7–78.6; 201 patients in 4 groups from 2 studies); PAR/HDM group 49.06 ± 1.26 (95 % CI 46.6–51.5; 2255 patients in 6 groups from 2 studies); and ‘Mixed’ group 50.12 ± 0.52 (95 % CI 49.1–51.1; 4899 patients in 7 groups from 3 studies) Fig. 3.

**Fig. 4:**
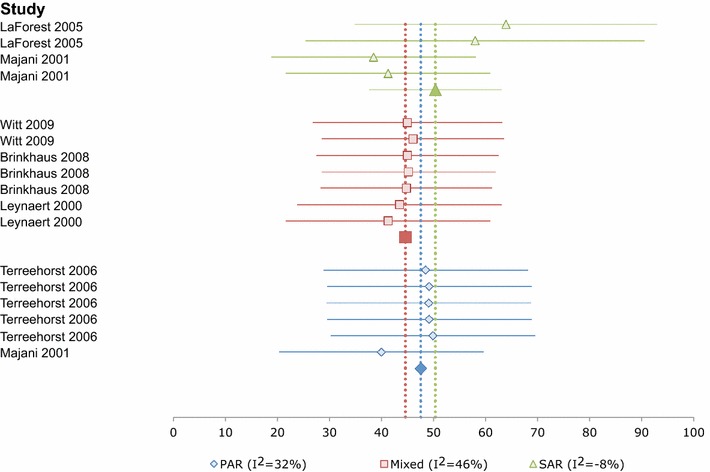
Forest plot of baseline SF-36/SF-12 mental component scores by phenotype, from 17 groups in six studies. 95 % confidence intervals for the Mixed and PAR summary measures are hidden by the *symbols*. Lower scores indicate poorer HRQL

Heterogeneity between SF-36 Mental Component Scores (MCS) was moderate/high when all studies were pooled (I^2^ = 73 %). When presented by phenotypic subgroups, there was no heterogeneity for the SAR/pollen group (I^2^ = −8 %), and heterogeneity was low both in the, ‘Mixed’ (I^2^ = 46 %) and PAR/HDM (I^2^ = 32 %) groups. Summary estimates for the MCS were: SAR/pollen group 50.38 ± 6.46 (95 % CI 37.7–63.0; 201 patients in 4 groups from 2 studies); PAR/HDM group 47.58 ± 1.28 (95 % CI 45.1–50.1; 2255 patients in 6 groups from 2 studies); and ‘Mixed’ group 44.62 ± 0.39 (95 % CI 43.9–45.4; 4899 patients in 7 groups from 3 studies) Fig. 4.

### Comparison of RQLQ total scores between SAR/pollen and PAR/HDM phenotypes

**Fig. 5:**
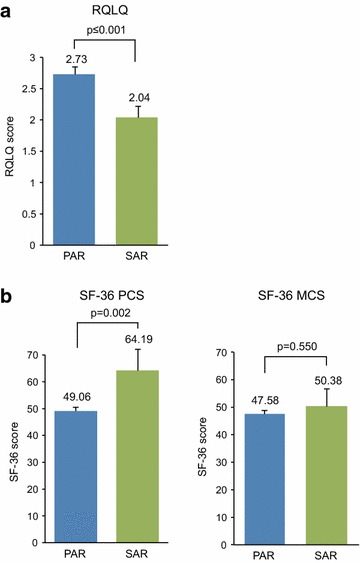
Overall RQLQ and SF-36 physical and mental component scores for the PAR and SAR phenotypes. **a** RQLQ scores of the PAR and SAR phenotypes; **b** *left* quality of life (QoL) measured by PCS of PAR and SAR patients. *Right* QoL measured by MCS of PAR and SAR patients. *Error bars* represent 95 % CI. Higher RQLQ scores and lower SF-36 scores indicate lower HRQL

ANOVA indicated that the RQLQ summary estimates of all three groups were different (F = 3.53; p = 0.024), but that the Mixed and PAR/HDM groups were not different (F = 0.134; p = 0.134). RQLQ summary scores for the SAR/pollen and PAR/HDM groups were different (F = 35.9; p ≤ 0.001), with the overall HRQL of PAR/HDM patients worse than that of SAR/pollen patients (2.73 ± 0.12 vs. 2.04 ± 0.18; p ≤ 0.001) Fig. 5a.

### Comparison of SF-36 physical and mental component scores between SAR/pollen and PAR/HDM phenotypes

SF-36 PCS summary estimates were different between the three groups (F = 5.75; p = 0.017), with no difference between the Mixed and PAR/HDM group (F = 0.857; p = 0.355). The SF-36 PCS summary of the PAR/HDM and SAR/pollen groups was different (F = 9.867; p = 0.002), with lower HRQL in the patients with PAR/HDM allergy vs. SAR/pollen allergy. Figure 5b shows that the SF-36 PCS of patients in the PAR/HDM group was significantly worse than that of those in the SAR/pollen group (49.06 ± 1.26 vs. 64.19 ± 7.89; p = 0.002).

SF-36 MCS summary scores for the three groups were also different (F = 4.95; p = 0.007), with differences between the Mixed and the PAR/HDM groups (F = 8.169; p = 0.004), but not between the SAR/pollen and PAR/HDM groups (F = 0.357; p = 0.550), indicating that HRQL in both groups was equally affected (47.58 ± 1.28 vs. 50.38 ± 6.46, p = 0.550). Figure 5b.

### Baseline RQLQ and SF-36 domain scores

**Fig. 6:**
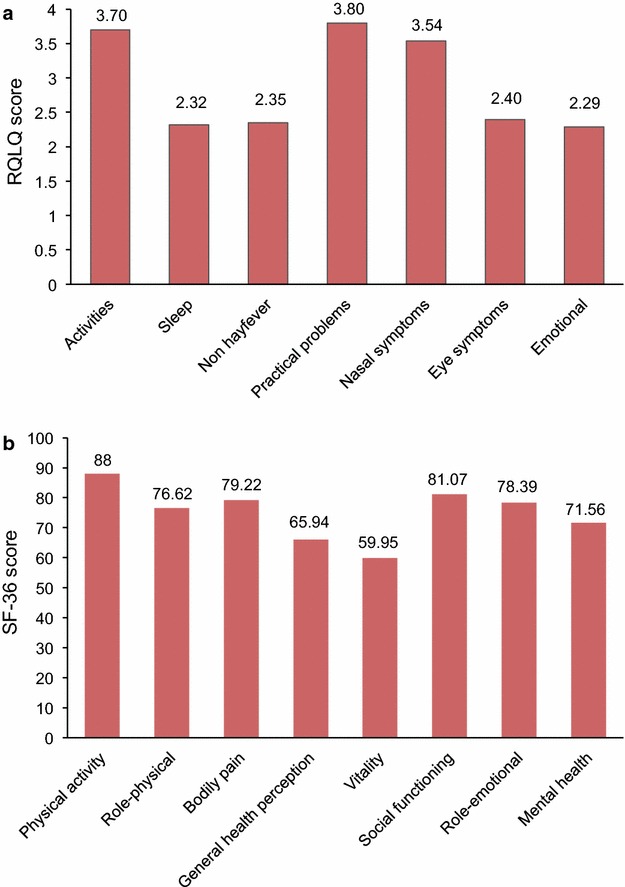
RQLQ and SF-36 domain scores. **a** RQLQ domain scores in the combined PAR/SAR population; **b** SF-36 domain scores in the PAR/SAR population

Eight studies [5, 47, 51–53, 55, 56, 58] reported RQLQ domain scores, or variants thereof. The ‘activities’ domain showed the highest heterogeneity (I^2^ = 76 %), with little or no heterogeneity (I^2^ = 9 %) in ‘non-hayfever symptoms’, or ‘emotional problems’ (I^2^ = 18 %), and medium heterogeneity (I^2^ = 31–35 %) in the remaining domains (Additional file 1: Figure S1). ‘Practical problems’ and ‘activities’ were the most affected domains, with the ‘emotional’ domain being the least affected (Fig. 6a).

Four studies [13, 14, 62, 63] reported SF-36 data by domain. The ‘role-emotional’ domain showed the highest heterogeneity (I^2^ = 43 %), with little or no heterogeneity in ‘physical activity’, ‘bodily pain’, ‘general health perception’, ‘vitality’ or ‘mental health’. (I^2^ = 0 % for all; Additional file 1: Figure S2). ‘Vitality’ was the most affected domain, with ‘physical activity’ being the least affected domain Fig. 6b. There were insufficient data to compare domain scores by allergy phenotype.

### Financial burden of AR and AA

Eleven studies were included in the analysis of direct and indirect costs; nine studies reported data for countries of interest [24, 62, 65–71] and two studies [72, 73] reported information for multiple countries using France and the United Kingdom as reference countries for calculating PPP costs (see Tables 1, 2). No studies that reported AR costs by severity were identified. One of the 17 studies included in the cost analysis was a randomized, controlled trial [72], and may not reflect costs in a real clinical practice setting.

**Table 1:** Direct costs

Country	Ref.	Age group	Direct costs			Values			
			Disease	Allergen	Definition	Country currency	Year of currency		PPP (2013 Int$)
Denmark	[65]	Adult	AR	SAR/grass	GP visit/extra GP visit/acute ward visit/loratadine/budesonide/fluticasone/salbutamol	307.49	2005	(€)	47.19
	[66]	Adult	AR	Mixed	Healthcare	1964.00	2002	(DKK)	300.96
France	[67]	Adult	AR	PAR/HDM	Desloratidine/medication (comorbidity)/medication (additional PAR)/GP visit/comorbidity GP visit	373.32	2007	(€)	492.39
	[68]	Adult	AR	SAR/grass	Direct costs	694.95	2003	(€)	261.64
				PAR/HDM	Direct costs	183.57	2003	(€)	130.71
		Children		SAR/grass	Direct costs	140.33	2003	(€)	200.01
				PAR/HDM	Direct costs	279.33	2003	(€)	398.12
Germany	[62]	Adult	AR	Mixed	GP visit/medication (unspecified)/hospitalization	771.12	2009	(€)	993.71
	[65]	Adult	AR	SAR/grass	GP visit/Extra GP Visit/Acute ward visit/loratadine/budesonide/fluticasone/salbutamol	383.85	2005	(€)	493.88
	[69]	Adult	AR	SAR/grass	Direct costs	356.37	1991	(DEM)	351.56
			AR	PAR/HDM	Direct costs	517.94	1991	(DEM)	884.64
			AR+AA	Mixed	Direct costs	544.02	1991	(DEM)	887.16
Italy	[70]	Adult	AR+AA	Mixed	Medication (unspecified)/outpatient care/emergency department/hospitalization	1589.30	2004	(€)	2568.52
	[71]	Children and adolescent	AR	SAR/grass	Direct costs	500.00	2003	(€)	826.75
				PAR/HDM	Direct costs	518.00	2003	(€)	856.51
				Mixed	Direct costs	506.00	2003	(€)	836.67
Multiple (1)	[72]	Adult	AR	PAR/HDM	GP visit/medication (unspecified)	100.44	2002	(€)	149.07
Multiple (2)	[73]	Adult	AR+AA	SAR/grass	Acute ward visit/asthma medication/budesonide/DESLORATIDINE/Extra GP or specialist visit/fluticasone/GP visit/medication (unspecified)/salbutamol	289.88	2005	(£)	527.27
Sweden	[65]	Adult	AR	SAR/grass	GP visit/extra GP visit/acute ward visit/loratadine/budesonide/fluticasone/salbutamol	595.80	2005	(€)	838.42

*DKK* Danish Krone, *DEM* Deutsche Mark, *Multiple (1)* Belgium, France, Germany, Italy, Spain, *Multiple (2)* United Kingdom, Germany, the Netherlands, Denmark, Sweden, Spain and Austria

**Table 2:** Indirect costs

Country	Ref.	Age group	Disease		Indirect costs		Values		
			Disease	Allergen	Definition	Country currency	Year of currency		PPP (2013 Int$)
Denmark	[65]	Adult	AR	SAR/grass	Absenteeism	667.36	2005	(€)	102.26
	[66]	Adult	AR	Mixed	Patient/societal	30606.00	2002	(DKK)	4689.98
France	[67]	Adult	AR	PAR/HDM	Absenteeism/presenteeism/Wage per work day	1480.68	2007	(€)	1952.95
	[68]	Adult	AR	SAR/grass	Indirect costs	210.86	2003	(€)	229.67
			AR	PAR/HDM	Indirect costs	161.14	2003	(€)	300.52
Germany	[62]	Adult	AR	Mixed	Indirect costs	765.84	2009	(€)	1073.38
	[65]	Adult	AR	SAR/grass	Absenteeism	352.04	2005	(€)	523.04
Italy	[71]	Children and adolescent	AR	SAR/grass	Indirect costs	2222.00	2003	(€)	3674.08
				PAR/HDM	Indirect costs	2057.00	2003	(€)	3401.25
				Mixed	Indirect costs	2166.00	2003	(€)	3581.48
Multiple (1)	[72]	Adult	AR	PAR/HDM	Absenteeism/presenteeism/Inability to perform usual daily activities/restriction over usual daily activities	4160.40	2002	(€)	6174.94
	[24]	Adult	AR	PAR/HDM	Absenteeism/presenteeism	1826.88	2001	(€)	2711.49
Multiple (2)	[73]	Adult	AR+AA	SAR/grass	Absenteeism/presenteeism/production loss	983.17	2005	(£)	1788.29
Sweden	[65]	Adult	AR	SAR/grass	Absenteeism	384.62	2005	(€)	50.68
Netherlands	[65]	Adult	AR	SAR/grass	Absenteeism	810.12	2005	(€)	1140.01
UK	[65]	Adult	AR	SAR/grass	Absenteeism	408.41	2005	(€)	742.85

*DKK* Danish Krone, *Multiple (1)* Belgium, France, Germany, Italy, Spain, *Multiple (2)* United Kingdom, Germany, the Netherlands, Denmark, Sweden, Spain and Austria

Direct comparison of total direct and indirect costs was complicated by differences in cost assessment in each study. Within countries, specific categories of costs (e.g. GP/specialist/visits, hospitalizations, medications) varied across studies, but we found very limited or no evidence for a trend of increasing or decreasing direct and indirect costs. The majority of the direct costs for AR were borne by the GP services; for patients with AR + AA, the AA part of the costs concerned medication and hospitalization costs. Medication costs, excluding over-the-counter medications, remained low Table 1.

The majority of the cost burden was indirect (Tables 1, 2) and caused by high absenteeism and presenteeism. Due to the lack of data, it was not possible to determine which had the higher cost; however, based on categories within each country, both were generally greater than the direct costs.

## Discussion

Grouping studies by seasonal or perennial symptoms gave an acceptable level of heterogeneity (I^2^ ≤ 63 %) that allowed summary estimates of HRQL from RQLQ and SF-36 instruments to be determined. Heterogeneity was generally higher for the SAR/pollen phenotype than for PAR/HDM, which could reflect the different types of baseline measurements in the selected studies, or the large heterogeneity of SAR. Patients with PAR/HDM allergy had poorer HRQL than those with SAR/pollen allergy when measured by RQLQ, SF-36 total score, and SF-36 PCS. The underlying reason for this difference is unclear, and could be due to differences in symptom frequency, duration, or severity. In Northern Europe, the pollen season is largely confined to the summer months, with the season for grass pollen, which is by far the most frequent cause of pollinosis, usually lasting from mid-May to July [74]; at the height of the pollen season, patients with SAR suffer symptoms approximately 50 % of the time and report an impact on their HRQL [75]. It can be hypothesized that the year-round nature of symptoms might be responsible for the observed poorer HRQL in PAR/HDM allergy; however, published studies report varying results. Bousquet found that the severity of AR was more important in determining HRQL, sleep, daily activities, and work performance than symptom duration [3]. Similar findings were reported by Delgado and colleagues, who observed that HRQL was lower in Spanish patients with olive or grass pollen allergy vs. HDM allergy. This was attributed to the greater severity of symptoms caused by the explosive nature of olive pollination, resulting in sudden and elevated pollen concentrations, in contrast to the more constant concentrations of perennial allergens that were more tolerable for patients [76]. Another study reported no significant differences in baseline RQLQ scores between patients with HDM, grass pollen, and *Parietaria* pollen allergy [48].

Analysis of SF-36 component scores showed that mental and physical component scores were affected in seasonal and perennial allergy, with HDM/PAR having a greater impact on physical vs. mental component scores. This supports the findings of other studies that report significant emotional problems and poor mental wellbeing in patients with AR, in addition to physical symptoms [13, 33, 77].

Meta-analyses were performed using HRQL measures from generic and disease-specific instruments. Generic questionnaires, like the SF-36, measure a wide range of physical, mental and psychosocial functions; they can be used in different health conditions and in the general population, and have the advantage of allowing comparisons of burden of illness across different disorders. Disease-specific instruments like the RQLQ more accurately reflect the patient’s inherent disease-associated problems, but scores cannot be directly compared with other populations. Both the generic and RQLQ instruments show improvements in HRQL in patients treated for AR; disease-specific questionnaires tend to show more important improvements, as they focus on the areas that are impacted by the disease [32]. Nowadays, HRQL questionnaires are used almost routinely in clinical studies of allergy treatments. Observed changes in HRQL generally reflect clinical outcomes; however, some studies report changes in HRQL that are not captured by symptom scores. Therefore, monitoring patients’ HRQL in clinical practice has been suggested as a possible strategy to improve the management of AR [78].

**Fig. 7:**
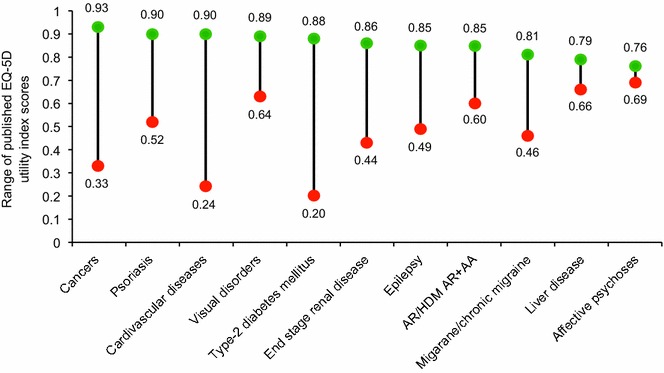
Range of EQ-5D utility index scores for selected chronic conditions. *Green* and *red circles* represent the upper and lower values, respectively. Values were obtained from published studies in populations with cancer [82], psoriasis [82], cardiovascular diseases [82], visual disorders [82], type-2 diabetes mellitus [82], end stage renal disease [82], epilepsy [83], AR/HDM or AR and AA [23, 84], migraine [84] or chronic migraine [85], liver disease [82], and active psychoses [84, 86]. Lower EQ-5D values indicate lower HRQL

Our analysis does not permit comparison of the HRQL of AR patients with that of the general population; however, a Spanish cross-sectional study that investigated the overall HRQL of patients with AR using SF-12 reported that HRQL was 25 % lower than in the general population [79]. Poor HRQL in patients with AR is also supported by evidence that successful treatment of AR symptoms with AIT [21–23] or symptomatic treatment [24–31] improves patient’s HRQL scores. A comparison of published EQ-5D utility baseline scores indicated that perennial allergy and/or AA (13 patients with AR and 12 with AR+AA), on a typical day with allergy symptoms, had a score of 0.60 [23], indicating comparable HRQL to diabetes (0.83) [80], cardiovascular disorders (0.73), musculoskeletal disorders (0.63), and psychosomatic disorders (0.57), when measured with the same EQ-5D instrument [81]. Ranges of published EQ-5D scores for AR and/or AA and other diseases are summarized in Fig. 7, and indicate that the impact of AR and/or AA on generic HRQL on a day with allergen exposure is not trivial, with an effect that is comparable to chronic conditions, such as liver disease, epilepsy, migraine, and visual disorders [23, 82–86].

There were insufficient data in the present analysis to compare HRQL in patients with AR vs. those with both AR and AA. Delgado and colleagues reported that HRQL associated with AR was worse in patients diagnosed with both AR and asthma than in those with only AR [76]. Using the SF-36 instrument, Leynaert reported that patients with asthma experienced greater impairments in physical functioning that those with AR alone, but asthma did not further impair mental wellbeing of patients with AR [13].

The cost analysis shows variations in direct and indirect AR costs between countries, which may result from true differences in AR costs or from differences in the health systems, diagnostic behavior, or reporting between countries. Indirect costs make up the majority of the cost burden of AR, in line with the outcomes of the most recent European [39] and US studies [34]. A few studies have reported higher direct vs. indirect costs [35, 87]; however, many studies estimate indirect costs by multiplying patients’ salaries by the amount of time they are absent from work due to AR. Health impairments associated with AR are often not severe enough to cause absence from work, although they can interfere with cognitive functioning, resulting in fatigue and an impaired ability to learn, concentrate, and make decisions [88], all of which can affect performance in the workplace. In one study, more than a third of patients with AR (36 %) reported reduced workplace performance, or ‘presenteeism’, which was higher than among patients with asthma (19 %) [89]. In the US, where up to 60 million people are affected by AR [90], annual productivity loss associated with a diagnosis of AR was estimated at $601 million in 1995. When the use of sedating allergy medications and workers’ self-assessments of their reduction work productivity due to AR were taken into account, the estimated annual at-work productivity losses increased to $2.4–$4.6 billion [37]. In 2006, the cost of lost productivity in the workplace due to AR was $593 per employee per year, and exceeded that of stress ($518), migraine ($277), depression ($273), and arthritis/rheumatism ($269) [36]. Comorbid AA resulted in higher costs than AR alone [91], or AR with non-allergic asthma [92], indicating its importance in driving costs. The cost of productivity loss is greater than the cost of treating AR symptoms; however, many cost analysis studies do not consider the cost to the patient of over-the-counter AR medications, which could lead to an underestimation of direct costs [34].

Some limitations of the study design should be acknowledged. Search terms were chosen from the RQLQ and SF-36 questionnaires, or were based on the suspected impact of AA and AR on patients’ HRQL. The articles identified by the search were mainly safety or efficacy studies that included HRQL as a secondary endpoint. Consequently, abstracts that did not include secondary outcomes would not be identified by the search. This could introduce a bias, such that only studies reporting a change in HRQL were identified. Inclusion of patients with comorbid AA in some groups could also affect HRQL scores, although we estimate any effect to be small. Moreover, the study only included HDM, and not pet allergens, as perennial allergens for perennial allergic rhinitis/asthma. Many of the studies from which data are collated were randomized controlled interventional studies, which may tend to include patients with more severe symptoms.

## Conclusions

This review presents summary data on HRQL levels in patients with allergic respiratory disease that may be used for comparison with assessments from individual studies. HRQL in PAR/HDM patients was significantly worse than that of SAR/pollen patients, when measured by both disease-specific and generic HRQL instruments, and was reflected by an impact on both physical and mental health. SAR/pollen and PAR/HDM affected MCS to a similar degree, despite a greater impact of PAR/HDM on PCS. A comparison of published EQ-5D utility index scores showed that the impact of PAR, with or without AA, on patients’ HRQL was comparable to other chronic diseases. The economic burden of AR is considerable, with indirect costs due to absenteeism and presenteeism making up the majority of costs. Greater awareness of the detrimental effects of AR on HRQL and its cost burden might encourage early diagnosis and treatment in order to minimize the disease burden and ensure beneficial and cost-effective outcomes.

## Additional file

10.1186/s12948-016-0049-9 Database search strategies;** Figure S1.** RQLQ domain scores by allergy phenotype. Studies are listed from the top as SAR, Mixed, and PAR; Figure S2 SF-36 domain scores by allergy phenotype. Studies are listed from the top as SAR, Mixed, and PAR.

## Abbreviations

AA: allergic asthma
AIT: allergy immunotherapy
AR: allergic rhinitis
ARIA: allergic rhinitis and its impact on asthma
GP: general practitioner
HDM: house dust mite
HRQL: health-related quality of life
MCS: mental component score
PAR: perennial allergic rhinitis
PCS: physical component score
PPP: purchasing power parities
RQLQ: rhinitis quality of life questionnaire
SAR: seasonal allergic rhinitis
USD: United States Dollars
