# Supplementary material for: Burden of allergic respiratory disease: a systematic review
Source: Clin Mol Allergy. 2016 Sep 28;14:12. doi: 10.1186/s12948-016-0049-9 (PMC5041537; doi:10.1186/s12948-016-0049-9)

Supplementary information

Database search strategies

Table S1 Ovid search strategy for National Institute For Health Research Health Technology Assessment Programme databases and outcomes

Topic	#	Term	Number of results				
			**Medline (30/01/14)**	**EBM (30/01/14)**	**Embase (30/01/14)**	**EconLit (30/01/14)**	**ALL sources (30/01/14)**
Population	1	Allerg*	160125	20449	171126	152	391146
	2	Rhin*	65944	13223	217391	27	154391
	3	Asthma.hw,mp	132121	7406	80643	178	324224
	4	1 and 2	25550	5184	33406	5	64224
	5	1 and 3	37049	3787	64950	4	105934
	6	4 or 5	51605	7814	79623	9	139213
Outcome	7	quality of life	185199	32860	297219	2083	518252
	8	Fatigue	65124	8331	137058	150	211021
	9	Impair*	449149	22880	595645	1070	1070743
	10	Dyspn*	38455	3247	92773	2	134715
	11	Dysphor*	3673	703	7062	2	11448
	12	Activ*	3607584	102537	3964291	73587	7757535
	13	Emotion*	126503	7715	163588	1962	300197
	14	Cogniti*	249739	24698	351688	4511	631702
	15	Eye	269749	12225	268689	559	551756
	16	Nasal*	93129	8665	87747	2	189739
	17	Nose	50216	2611	89187	17	142237
	18	practical	164772	4585	164384	10180	344405
	19	Functional	2538295	88797	3007084	59843	5702092
	20	Impact*	529644	39581	711237	102616	1386072
	21	Sleep	122791	12884	152196	107	288389
	22	Work	580606	20092	672675	51176	1326717
	23	School	156849	10900	386544	20428	575717
	24	Cost	312480	43218	500688	71907	929426
	25	Prevalence	424497	14927	567133	2529	1010896
	26	Incidence	571991	42823	660675	13599	1290802
	27	7 or 8 or 9 or 10 or 11 or 12 or 13 or 14 or 15 or 16 or 17 or 18 or 19 or 20 or 21 or 22 or 23 or 24 or 25 or 26	7851421	314447	8989198	336384	17514992
HDM OR GRAS	28	dust mite*	5355	667	7105	0	13147
	29	house adj2 mite*	4519	585	5802	0	10922

	30	28 or 29	5459	676	7173	0	13328
	31	grass	14759	951	18258	324	34344
	32	pollen	22554	1890	24366	31	48910
	33	31 and 32	3012	782	5018	1	8816
	34	30 or 33	8079	1422	11425	1	20949
Papers	35	6 and 27 and 34	2737	702	4678	0	8124
	36	limit 35 to (humans and yr='2000-Current')	1492	403	2989	0	4892
	37	(editorial or letter or news or case reports).pt.	2795475	7217	1150854	0	3956545
	38	36 not 37	1461	403	2943	0	4815
	39	remove duplicates from 38	1455	395	2839	0	3095
	40	limit 39 to english language	1326	395	2581	0	2810
	41	Limit 41 to journal articles	1326	337	2581	0	2766

Additional search strategies and outcomes

National Guidelines Clearing House (http://guideline.gov/); Keyword: respiratory hypersensitivity; Clinical Specialty: Allergy and Immunology; Publication Years: 2000, 2002, 2003, 2004, 2005, 2006, 2007, 2008, 2009, 2010, 2011, 2012, 2013; 19 studies identified

NHS Evidence (http://www.evidence.nhs.uk/), no studies identified

EuroScan (http://euroscan.org.uk/); Technology Search: rhinitis; Speciality: Respiratory disease, immunotherapy; 1 study identified

ClinicalTrials.gov (http://www.clinicaltrials.gov) (allergic AND (rhinitis OR asthma)) AND (mites or grass); 8 studies identified.

HEED (Wiley InterScience) AX=allerg* AND AX=rhin* OR AX=allerg* AND AX=asthma AND TY= METHODOLOGICAL OR STUDY OR STUDIES OR REVIEW; 169 studies identified.

Figure S1 RQLQ domain scores by allergy phenotype. Studies are listed from the top as SAR, Mixed, and PAR. The scale below the lower plot applies to all plots in each column.

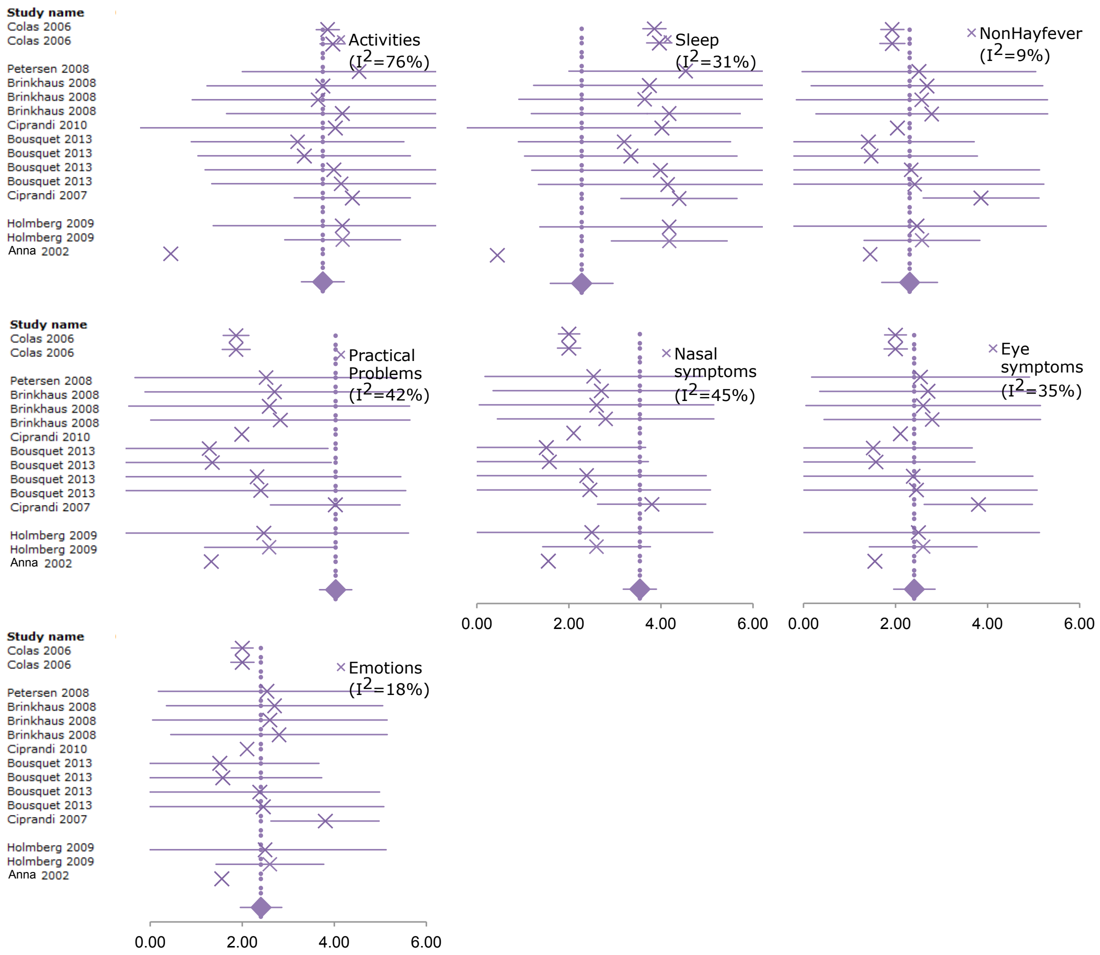

Figure S2 SF-36 domain scores by allergy phenotype. Studies are listed from the top as SAR, Mixed, and PAR. The scale below the lower plot applies to all plots in each column.

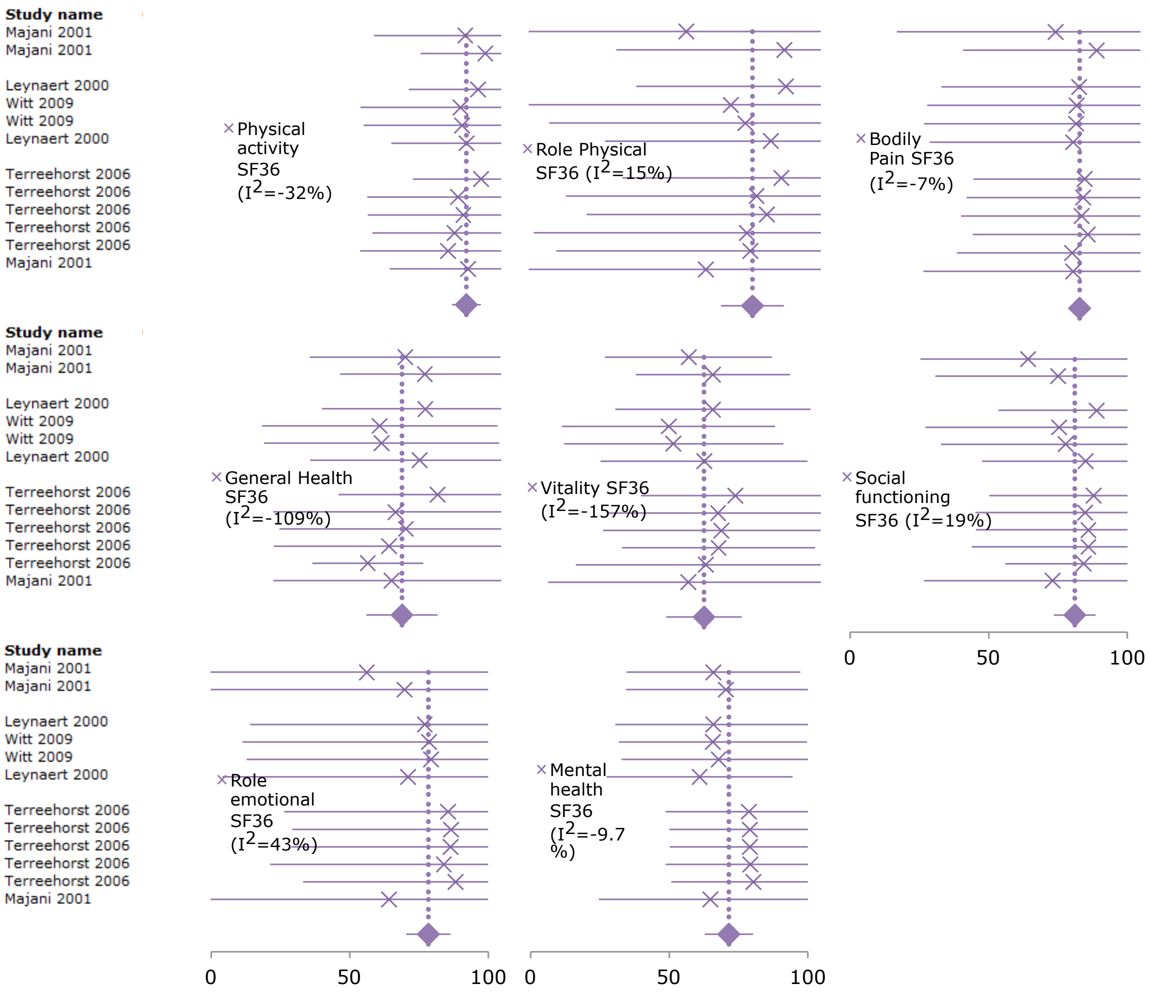

Supplement: Supplementary file 1 — 10.1186/s12948-016-0049-9 Database search strategies; Figure S1. RQLQ domain scores by allergy phenotype. Studies are listed from the top as SAR, Mixed, and PAR; Figure S2 SF-36 domain scores by allergy phenotype. Studies are listed from the top as SAR, Mixed, and PAR. [file 12948_2016_49_MOESM1_ESM.pdf]
